# Reclassification of moderate aortic stenosis based on data-driven phenotyping of hemodynamic progression

**DOI:** 10.1038/s41598-023-33683-1

**Published:** 2023-04-24

**Authors:** Iksung Cho, William D. Kim, Subin Kim, Kyu-Yong Ko, Yeonchan Seong, Dae-Young Kim, Jiwon Seo, Chi Young Shim, Jong-Won Ha, Makoto Mori, Aakriti Gupta, Seng Chan You, Geu-Ru Hong, Harlan M. Krumholz

**Affiliations:** 1 Division of Cardiology, Department of Internal Medicine, Severance Hospital, Yonsei University, 50-1, Yonsei-ro, Seodaemun-gu, Seoul, 03722 Korea; 2 Chung-Ang University College of Medicine, Seoul, Korea; 3 Division of Cardiac Surgery, Department of Surgery, Yale School of Medicine, New Haven, CT USA; 4 Center for Outcomes Research and Evaluation, Yale New Haven Hospital, New Haven, CT USA; 5 Department of Cardiology, Cedars-Sinai Medical Center, Los Angeles, CA USA; 6 Department of Biomedical Systems Informatics, Yonsei University College of Medicine, Seoul, Korea; 7 Institute for Innovation in Digital Healthcare, Yonsei University, 50-1, Yonsei-ro, Seodaemun-gu, Seoul, 03722 Korea; 8 Section of Cardiovascular Medicine, Department of Internal Medicine, Yale School of Medicine, New Haven, CT USA; 9 Department of Health Policy and Management, Yale School of Public Health, New Haven, CT USA

**Keywords:** Cardiology, Cardiovascular biology

## Abstract

The management and follow-up of moderate aortic stenosis (AS) lacks consensus as the progression patterns are not well understood. This study aimed to identify the hemodynamic progression of AS, and associated risk factors and outcomes. We included patients with moderate AS with at least three transthoracic echocardiography (TTE) studies performed between 2010 and 2021. Latent class trajectory modeling was used to classify AS groups with distinctive hemodynamic trajectories, which were determined by serial systolic mean pressure gradient (MPG) measurements. Outcomes were defined as all-cause mortality and aortic valve replacement (AVR). A total of 686 patients with 3093 TTE studies were included in the analysis. Latent class model identified two distinct AS trajectory groups based on their MPG: a slow progression group (44.6%) and a rapid progression group (55.4%). Initial MPG was significantly higher in the rapid progression group (28.2 ± 5.6 mmHg vs. 22.9 ± 2.8 mmHg, *P* < 0.001). The prevalence of atrial fibrillation was higher in the slow progression group; there was no significant between-group difference in the prevalence of other comorbidities. The rapid progression group had a significantly higher AVR rate (HR 3.4 [2.4–4.8], *P* < 0.001); there was no between-group difference in mortality (HR 0.7 [0.5–1.0]; *P* = 0.079). Leveraging longitudinal echocardiographic data, we identified two distinct groups of patients with moderate AS: slow and rapid progression. A higher initial MPG (≥ 24 mmHg) was associated with more rapid progression of AS and higher rates of AVR, thus indicating the predictive value of MPG in management of the disease.

## Introduction

Aortic stenosis (AS) is the most common valvular heart disease in developed countries and being a chronic and progressive disease, its prevalence is increasing as a result of population aging^1,2^. Patients with symptomatic severe AS experience high morbidity and mortality, and benefit from aortic valve replacement (AVR)^3^. In patients with asymptomatic moderate to severe AS, there have been recent attempts to reclassify AS based on cardiac dysfunction to improve risk stratification^4,5^. However, unlike severe symptomatic AS, the management and follow-up of moderate AS lacks consensus^1,5^. While some studies have suggested poor survival rates from moderate AS similar to those of severe AS^6^, others showed only slightly increased risk of death compared with mild AS^7^. The natural history of moderate AS and associated outcomes need to be better understood.

Latent class trajectory modeling (LCTM) is a statistical method that identifies specific groups of patients defined by serial measurement of observed variables, such as hemodynamic measurements^8,9^. Hence, phenotyping using a series of longitudinal hemodynamic parameters may help to identify risk factors for progression of AS and allow appropriate management strategies based on risk stratification in patients with moderate AS. This study sought to describe the progression to severe grade requiring AVR and outcomes in patients with moderate AS, while determining the clinical factors associated with progression using a trajectory-based method to detect different profiles of moderate AS.

## Methods

### Study cohort

Of the 218,958 patients who underwent transthoracic echocardiography (TTE) at Severance Hospital in Seoul, South Korea between January 2010 and February 2021, 2344 patients above 18 years of age were identified to have moderate AS (mean pressure gradient [MPG] ≥ 20 mmHg and < 40 mmHg). The following exclusion criteria were then applied: previous surgical AVR (n = 569) or transcatheter AVR (TAVR; n = 199), fewer than three TTE studies available (n = 855), less than 3 months of follow-up (n = 24), and limited access to data (n = 11). The final analysis included data for 686 patients with moderate AS at baseline and 3093 echocardiography studies (Supplementary Fig. 1). Additionally, patients with rheumatic heart disease or concomitant non-aortic valvular diseases were also excluded and all analysis were performed to the subgroup consisting of purely degenerative moderate AS patients (n = 311). The following valvular diseases were excluded: (1) moderate or severe mitral stenosis, (2) severe aortic or mitral or tricuspid regurgitation, and (3) bicuspid aortic stenosis. The study followed the Strengthening the Reporting of Observational Studies in Epidemiology reporting guideline^10^. The institutional review board approved the study (approval number 4-2021-0408) and waived the requirement for informed consent in view of the retrospective nature of the research.

### Transthoracic echocardiography

Comprehensive two-dimensional and Doppler TTE measurements were performed according to the current guidelines^11^. Peak velocity was measured from multiple acoustic windows of continuous-wave Doppler sonograms to obtain the highest maximal velocities^11^. A simplified Bernoulli equation was used to calculate peak pressure gradients (PPGs)^12^. The position yielding the highest aortic valve (AV) velocity was used to determine the time–velocity integral and to calculate the mean MPG using the average value for at least three traced signals. AV area was calculated using the continuity equation and indexed for body surface area^13^. Left ventricular (LV) stroke volume was calculated by multiplying the LV outflow tract area by the outflow tract time–velocity integral. The severity of AS was determined by MPG and valve area based on the current American College of Cardiology/American Heart Association guidelines^14^. The etiology of AS was determined according to valve anatomy and paravalvular structures and classified as a rheumatic, degenerative, or bicuspid AV. Changes in PPG, MPG, and AV area were calculated as the difference between baseline and follow-up measurements divided by the time interval between the two examinations. All TTE data were analyzed by experienced cardiologists who were blinded to all clinical data.

### Group classification using LCTM

LCTM was used to classify groups of AS with distinctive trajectories of hemodynamic status as determined by the MPG progression rate. LCTM classifies patients into groups based on similarity of patient-level trajectories using an unsupervised learning approach^15^. A detailed description of trajectory modeling and the selection process is shown in Supplementary Table 1. Models with 2–6 trajectories were considered for selection. Each trajectory was modeled using linear or quadratic MPG terms with or without controlling for age and sex. The appropriate model and its number of trajectories were selected by considering the following: the Bayesian information criterion value, resulting trajectory groups each containing at least 10% of patients, average posterior probability of each class of greater than 90%, relative entropy, and clinical interpretability^16,17^. The baseline clinical characteristics and echocardiographic findings were then compared among the different trajectories.

### Outcome measures and covariates

Progression of disease was evaluated with AVR rates during the follow-up period and additionally the time to events, being analyzed at 1, 2, and 5 years. Patient mortality was ascertained by linking to the Korean Ministry of the Interior and Safety public database, which stores the national mortality data. Patient demographics (age, sex, and body mass index), comorbidities (hypertension, dyslipidemia, diabetes, peripheral arterial occlusive disease, stroke, chronic kidney disease, chronic obstructive pulmonary disease, coronary artery disease, coronary artery bypass graft, congestive heart failure, atrial fibrillation, rheumatic heart disease, bicuspid AV, and aortic regurgitation), were obtained from physician notes, and echocardiographic findings (initial MPG, initial PPG, initial peak aortic velocity (PV), initial AV area, time–velocity integral, LV ejection fraction, and left ventricle end-systolic/diastolic dimensions) were abstracted from echocardiographic reports, and compared between the two groups.

### Defining clinical and echocardiographic factors for trajectory groups allocation

Receiver operating characteristic (ROC) curve analysis was performed to establish the value of MPG as a predictor of group classification. We identified the cut-off value for MPG that demonstrated the best sensitivity and specificity using the ROC curve as reference. Logistic regression was used to assess the association between baseline characteristics and group classification while using the posterior probabilities of class membership as the weight. Patient demographics (age and sex), comorbidities (hypertension, dyslipidemia, diabetes, peripheral arterial occlusive disease, stroke, chronic kidney disease, chronic obstructive pulmonary disease, coronary artery disease, coronary artery bypass graft, congestive heart failure, atrial fibrillation, rheumatic heart disease, bicuspid AV, and aortic regurgitation), and initial MPG were used as variates.

### Statistical analysis

Descriptive statistics were used to characterize baseline characteristics, comorbidities, and echocardiographic findings. Categorical variables are presented as percentages and continuous variables as the mean and standard deviation. *P*-values were obtained using either the two-sided *t* test or the Chi-square test. Incidence rates for mortality and AVR (overall, surgical, and transcatheter) were calculated by dividing the number of events by total follow-up duration (person-time), with univariate analyses using Cox proportional hazard models being applied to estimate the hazard ratios (HRs) for comparison between groups. Kaplan–Meier curves were used to calculate survival and AVR-free probability; between-group differences were analyzed using the log-rank test. The association between the MPG value and each outcome measure was evaluated using restricted cubic spline curves. Multivariate Cox regression was used to assess the independent association between the trajectory group and the outcome with adjustment for factors identified to be related to the outcome in univariate analysis. All analyses were performed using R version 3.9 (R Foundation for Statistical Computing, Vienna, Austria). LCTM was performed using LCTMtools package (https://rstudio-pubs-static.s3.amazonaws.com/522393_3aa7f65898f8426e9c0a92d7971b619d.html). A two-sided *P*-value < 0.05 was considered statistically significant.

## Results

### Baseline characteristics

The baseline characteristics of the 686 patients included in this study are shown in Table 1. The mean patient age was 67.8 ± 12.4 years, and 361 patients (52.6%) were women. A history of hypertension was found in 62.8% of the patients and 11.4% had a history of congestive heart failure. Rheumatic heart disease was the cause of AS in 144 patients (21.0%) and bicuspid aortic valve in 74 (10.8%). Concomitant aortic regurgitation of at least moderate grade was present in 22.1% of the patients. During the follow-up period of 4.8 ± 2.7 years, the median (IQR) number of echocardiography studies was 4 (3–5), with a mean interscan interval of 1.1 ± 0.6 years. The reason for index study was mostly cardiac evaluation for systematic disease or other cardiac disease (46.4%), followed by symptoms of AS (29.2%), routine follow-up for known mild AS (22.7%), and cardiac evaluation for non-cardiac procedures (1.7%).

**Table 1:** Baseline demographic and clinical characteristics.

Characteristic	Overall (n = 686)	Slow progression group (n = 306)	Rapid progression group (n = 380)	*P-*value
Age, years	67.8 ± 12.4	67.0 ± 13.0	68.5 ± 11.8	0.139
Female (%)	361 (52.6)	164 (53.6)	197 (51.8)	0.648
BMI > 25 kg/m^2^ (%)	239 (34.8)	106 (34.6)	133 (35.0)	0.922
Comorbid conditions (%)				
Hypertension	431 (62.8)	200 (65.4)	231 (60.8)	0.218
Dyslipidemia	246 (35.9)	118 (38.6)	128 (33.7)	0.214
Diabetes	230 (33.5)	112 (36.6)	118 (31.1)	0.126
PAOD	32 (4.7)	11 (3.6)	21 (5.5)	0.233
Stroke	67 (9.8)	36 (11.8)	31 (8.2)	0.114
Chronic kidney disease	123 (17.9)	61 (19.9)	62 (16.3)	0.219
COPD	26 (3.8)	12 (3.9)	14 (3.7)	0.871
Coronary artery disease	181 (26.4)	85 (27.8)	96 (25.3)	0.458
Coronary artery bypass graft	30 (4.4)	14 (4.6)	16 (4.2)	0.816
Congestive heart failure	78 (11.4)	46 (15.0)	32 (8.4)	0.007
Atrial fibrillation	168 (24.5)	91 (29.7)	77 (20.3)	0.004
Rheumatic heart disease	144 (21.0)	73 (23.9)	71 (18.7)	0.098
Bicuspid aortic valve	74 (10.8)	26 (8.5)	48 (12.6)	0.083
Aortic regurgitation	155 (22.6)	68 (22.2)	87 (22.9)	0.834
Reason for echocardiography				
Symptomatic*	200 (29.2)	94 (30.7)	106 (27.9)	0.419
Routine follow-up for known mild AS	156 (22.7)	53 (17.3)	103 (27.1)	0.002
Cardiac evaluation for systematic disease or other cardiac disease^†^	318 (46.4)	152 (49.7)	166 (43.7)	0.118
Cardiac evaluation for non-cardiac procedures/surgery	12 (1.7)	7 (2.3)	5 (1.3)	0.335
Echocardiographic findings				
Mean interscan interval, years	1.1 ± 0.6	1.2 ± 0.6	1.1 ± 0.6	0.641
Initial MPG, mmHg	25.8 ± 5.2	22.9 ± 2.8	28.2 ± 5.6	< 0.001
MPG mean progression rate, mmHg/year	2.5 ± 4.8	0.3 ± 3.6	4.2 ± 4.9	< 0.001
Initial PPG, mmHg	45.3 ± 9.6	40.5 ± 5.9	49.2 ± 10.2	< 0.001
Initial peak velocity, m/s	3.1 ± 1.1	2.7 ± 1.3	3.4 ± 1.0	< 0.001
Initial AVA by CE, cm^2^	1.2 ± 0.2	1.3 ± 0.2	1.1 ± 0.2	< 0.001
LVOT VTI, cm	24.0 ± 5.2	23.5 ± 4.8	24.4 ± 5.5	0.026
LVEF (%)	65.3 ± 10.7	63.6 ± 12.3	66.7 ± 9.0	< 0.001
LVEF < 40% (%)	18 (2.6)	13 (4.2)	5 (1.3)	0.017
LV end-diastolic dimension, mm	50.2 ± 6.4	50.9 ± 6.8	49.6 ± 6.0	0.009
LV end-systolic dimension, mm	33.0 ± 6.6	34.0 ± 7.6	32.1 ± 5.5	< 0.001

*AVA* aortic valve area, *BMI* body mass index, *CE* continuity equation, *COPD* chronic obstructive pulmonary disease, *LV* left ventricular, *LVEF* left ventricular ejection fraction, *LVOT* left ventricular outflow tract, *MPG* mean pressure gradient, *PAOD* peripheral arterial occlusive disease, *PPG* peak pressure gradient, *VTI* time–velocity integral.

*Symptomatic: Chest pain, dyspnea, syncope, dizziness, edema, general weakness.

^†^Systematic disease or other cardiac disease: Other cardiac disease (arrythmia, coronary artery disease, hypertrophic cardiomyopathy, infective endocarditis), systematic disease (chronic kidney disease, intracranial hemorrhage, sepsis).

### Classification of trajectory of moderate AS

**Figure 1:**
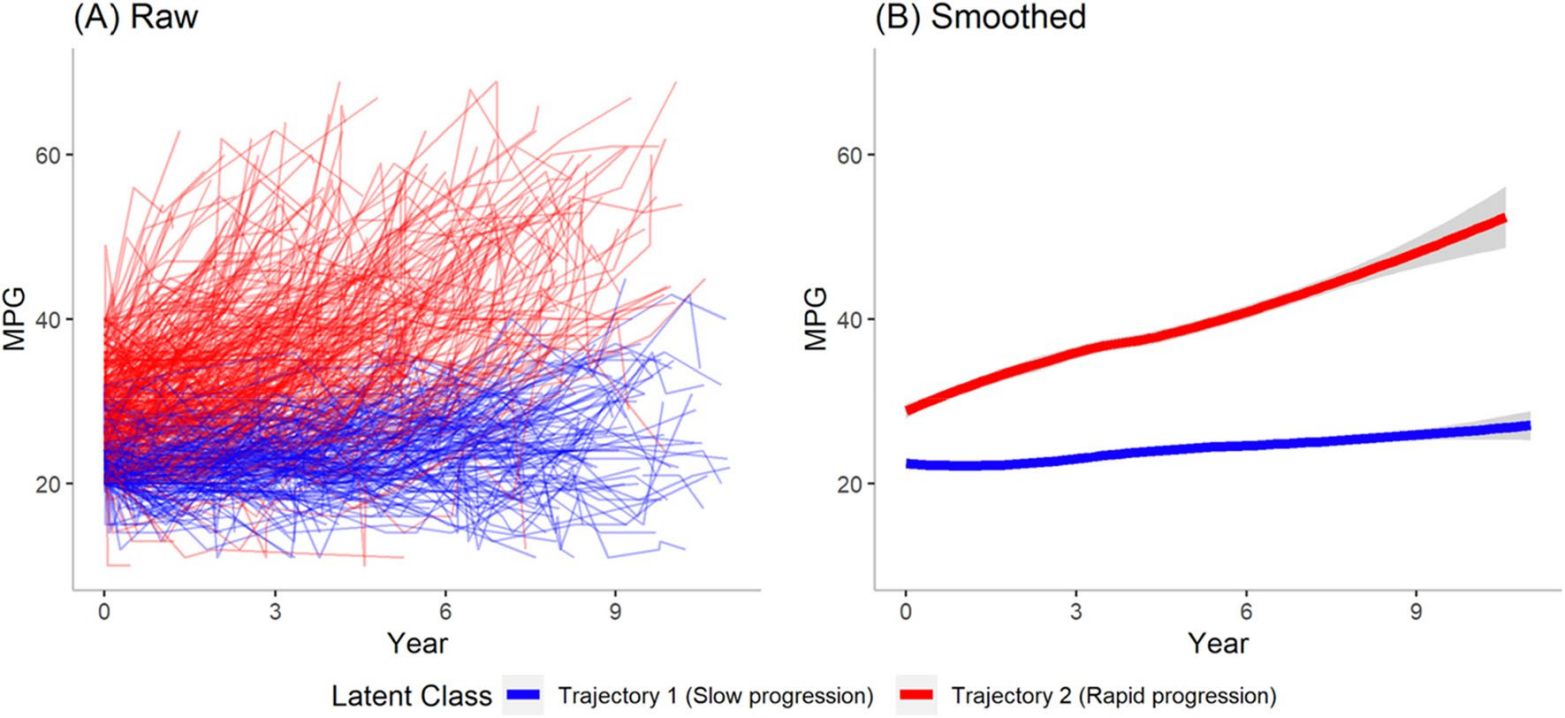
Classification of patients with moderate aortic stenosis using LTCM based on progression of MPG. (**A**) Raw LCTM results. (**B**) Smoothed LCTM results identify two distinct trajectories. *LCTM* latent class trajectory modeling, *MPG* mean pressure gradient.

The best fitting model in LCTM identified two trajectories of moderate AS according to progression of the MPG (Fig. 1). The parameters identified are compared with those using other models in Supplementary Table 2. Trajectory 1 was identified as a slow progression group (n = 306) and trajectory 2 as a rapid progression group (n = 380). The baseline characteristics and echocardiographic findings in the two groups are shown in Table 1. Overall, there was no statistically significant difference in comorbidities between the two groups, except for congestive heart failure and atrial fibrillation, which were more prevalent in the slow progression group. Compared to the slow progression group, the initial MPG was higher (28.2 ± 5.6 mmHg vs. 22.9 ± 2.8 mmHg, *P* < 0.001) and MPG progression was faster (4.2 ± 4.9 mmHg/year vs. 0.3 ± 3.6 mmHg/year, *P* < 0.001) in rapid progression group. When progression to severe AS was defined as having MPG > 40 mmHg during follow-up, there were a total of 267 patients (38.9%) that progressed to severe grade with significantly more patients in the rapid progression group (68.7% vs. 2.0%, P < 0.001).

A total of 165 moderate AS patients with 553 echocardiography results between March 2021 and December 2022 as a model validation group under identical inclusion and exclusion criteria. LCTM again identified two distinct trajectories with progression patterns similar to that of the main study group (Supplementary Fig. 2).

### Outcomes according to the trajectory group

**Figure 2:**
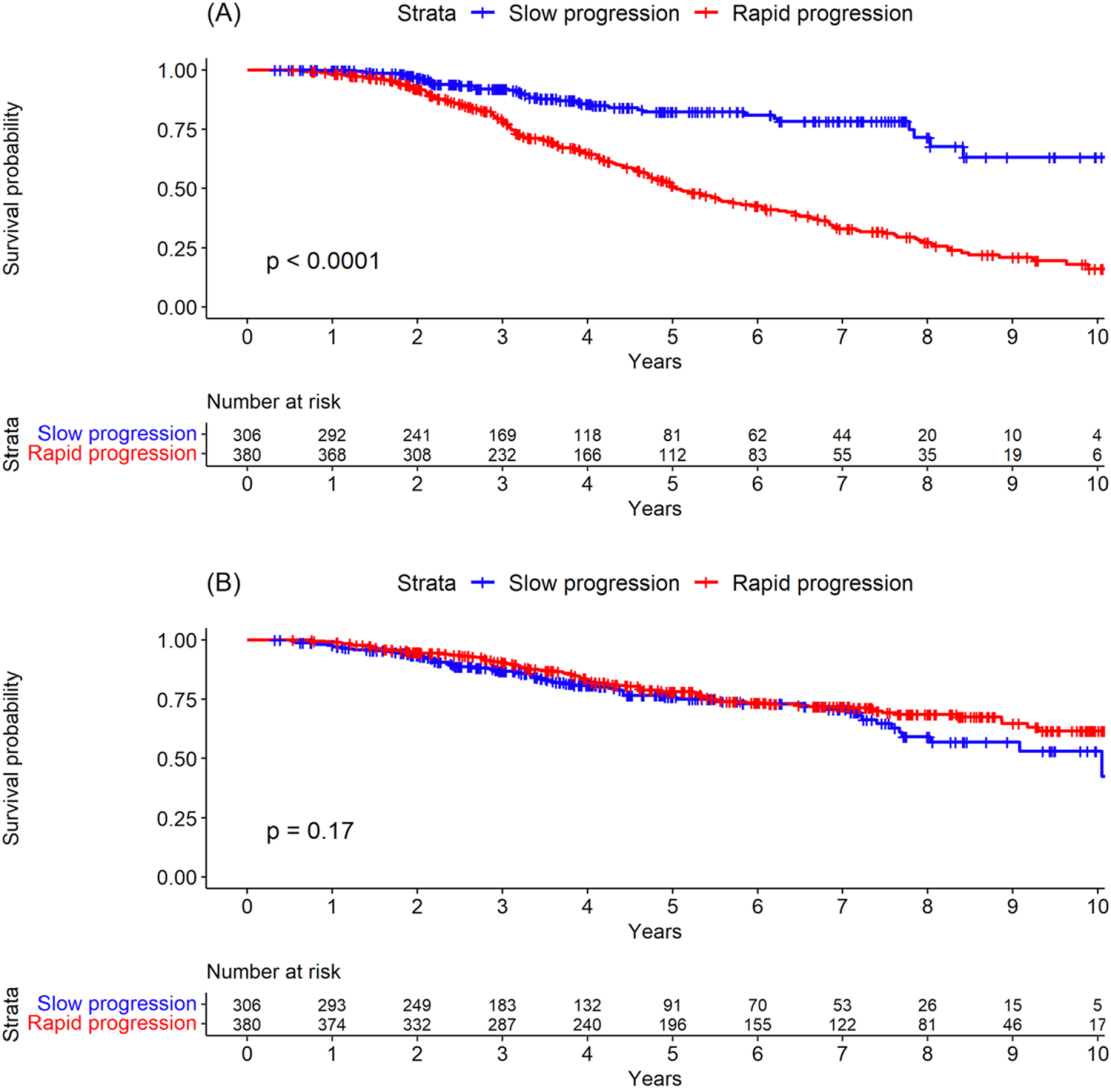
Kaplan–Meier curves for (**A**) aortic valve replacement and (**B**) death according to progression group.

The incidence rates for the defined outcomes in each group are shown in Table 2. The HRs for each outcome were adjusted with the covariates that were statistically significant in univariate Cox analysis (Supplementary Table 3). In total, 237 patients underwent AVR; there were significantly more cases in the rapid progression group than in the slow progression group (HR 3.4, 95% CI 2.4–4.8, *P* < 0.001; 12.4 cases per 100 person-years [95% CI 10.8–14.3] vs. 3.4 cases per 100 person-years [95% CI 2.5–4.6]). When analyzed by AVR method, the incidence rate was higher for both surgical AVR and TAVR in the rapid progression group (Table 2, Supplementary Fig. 3). The respective cumulative 1-year, 2-year, and 5-year AVR rates were 0.3%, 2.9%, and 11.1% in the slow progression group and 1.6%, 7.6%, and 37.4%, respectively, in the rapid progression group (Supplementary Table 4). Figure 2 shows the significant difference in AVR rates between the two groups (*P* < 0.001).

**Table 2:** Incidence rates and risk of each outcome in the two trajectory groups.

Parameters	Incidence rate (CI)		Hazard ratio (95% CI)*	
	Slow progression group	Rapid progression group		*P*-value
AVR	3.4 (2.5–4.6)	12.4 (10.8–14.3)	2.6 (1.8–3.8)^†^	< 0.001
Surgical	2.6 (1.7–3.6)	8.2 (6.8–9.6)	2.7 (1.7–4.2)^‡^	< 0.001
Transcatheter	0.8 (0.4–1.5)	3.1 (2.3–3.9)	3.0 (1.4–6.1)^§^	0.003
Mortality	5.4 (4.2–6.9)	4.7(3.8–5.7)	0.7 (0.5–1.0)^∥^	0.079

*AVR* aortic valve replacement, *CI* confidence interval.

*Hazard ratios for rapid progression group vs slow progression group.

^†^Adjusted with group type, age (10 years), peripheral arterial occlusive disease, chronic kidney disease, atrial fibrillation, rheumatic heart disease, initial mean pressure gradient.

^‡^Adjusted with group type, age (10 years), hypertension, dyslipidemia, coronary artery disease, coronary artery bypass graft, initial mean pressure gradient.

^§^Adjusted with group type, age (10 years), hypertension, dyslipidemia, diabetes, peripheral arterial occlusive disease, chronic kidney disease, coronary artery disease, coronary artery bypass graft, rheumatic heart disease, bicuspid aortic valve, aortic regurgitation.

^∥^Adjusted with group type, sex, age (10 years), hypertension, diabetes, peripheral arterial occlusive disease, stroke, chronic kidney disease, chronic obstruction pulmonary disease, coronary artery disease, coronary artery bypass graft, heart failure, rheumatic heart disease, bicuspid aortic valve, aortic regurgitation.

There were a total of 164 deaths during the follow-up period, with no between-group difference in the mortality rate (HR 0.7, 95% CI 0.5–1.0, *P* = 0.079; 5.4 deaths per 100 person-years in the slow progression group [95% CI 4.2–6.9] vs 4.7 deaths per 100 person-years in the rapid progression group [95% CI 3.8–5.7]) (Table 2, Fig. 2). The respective cumulative 1-year, 2-year, and 5-year mortality rates were 2.3%, 6.5%, and 17.6% in the slow progression group and 0.8%, 5.3%, and 18.2% in the rapid progression group, respectively (Supplementary Table 4).

### Initial clinical and echocardiographic factors associated with rapid progression group allocation

Given that the rapid progression group experienced AVR earlier and more frequently compared to slow progression group, it would be clinically useful to predict whether a patient diagnosed with moderate AS will fall into either the rapid or slow progression group. As depicted in Supplementary Fig. 4, an initial MPG of 24 mmHg showed the highest predictability as the cut-off value (area under ROC = 0.789) to determine rapid progression group. Further, univariate logistic regression analysis was performed to identify parameters that were significantly more common in the rapid progression group (Supplementary Table 5) and identified a significant positive correlation with an initial MPG ≥ 24 mmHg (odds ratio [OR] 6.36, 95% CI 3.97–10.30, *P* < 0.001). Age and sex were not associated with allocation to the rapid progression group; among the clinical characteristics, only atrial fibrillation (OR 0.60, 95% CI 0.37–0.99, *P* = 0.045) showed a negative correlation. Age-trajectory and sex-trajectory interactions were further analyzed; interactions between age and trajectory were statistically significant in AVR and surgical AVR, while sex-trajectory interaction showed no significance in all outcomes (Supplementary Tables 6 and 7). With adjustment of covariates, multivariate analysis revealed the initial MPG to be the sole independent positive predictor of being in the rapid progression group (OR 6.29, 95% CI 3.92–10.24, *P* < 0.001) (Supplementary Table 8).

Additional analysis from the model validation group (Supplementary Fig. 5) showed similar results with an initial MPG of 25 mmHg as the cut-off value (area under ROC = 0.832).

### MPG and risk of AVR and mortality

Supplementary Fig. 6 shows the association between MPG on a continuous scale and the risk of each outcome using cubic spline analysis. When an MPG of 24 mmHg was entered as a knot vector, determining the control point, there were increased risks of AVR with a higher MPG. However, there was no significant change in the risk of mortality when MPG increased or decreased.

### Results in subgroup of degenerative moderate AS patients

Overall, the subgroup of degenerative moderate AS patients showed similar results to that of the total study cohort. Two groups were identified by LCTM with a cutoff initial MPG value of 25 mmHg (Supplementary Fig. 7): (1) slow progression group (n = 231) and (2) rapid progression group (n = 80). The baseline demographics and clinical characteristics are presented in Supplementary Table 9, showing higher rates of dyslipidemia, diabetes, and congestive heart failure in the slow progression group. The rapid progression group had a significantly higher progression rate of MPG compared to the slow progression group (4.6 ± 5.8 mmHg/year vs. 1.5 ± 5.6 mmHg/year, P < 0.001). Outcomes of AVR and mortality again showed similar results; the rapid progression group showed higher rates of AVR (HR 3.0, 95% CI 1.9–4.6, P < 0.001; 16.7 cases per 100 person-years [95% CI 12.1–22.5] vs. 6.1 cases per 100 person-years [95% CI 4.5–8.1]), while there was no significant difference in mortality between the two groups (HR 1.4, 95% CI 0.9–2.3, P = 0.184; 6.8 deaths per 100 person-years in the slow progression group [95% CI 5.1–8.8] vs 6.4 deaths per 100 person-years in the rapid progression group [95% CI 4.0–9.6]) (Supplementary Table 10, Supplementary Fig. 8). Univariate Cox analysis for each outcome revealed that older age, a history of diabetes, chronic kidney disease, and coronary artery bypass graft were associated with higher mortality rates, while rapid progression group and a history of aortic regurgitation were associated with higher AVR rates (Supplementary Table 11). Finally, univariate logistic regression analysis was performed to identify parameters that were significantly more common in the rapid progression group (Supplementary Table 12), showing a significant positive correlation with an initial MPG ≥ 25 mmHg (odds ratio [OR] 16.70, 95% CI 5.24–77.40, P < 0.001).

## Discussion

In this study of 686 moderate AS cases with clinical and echocardiographic data available, we were able to identify two distinct trajectories of disease progression based on evaluation of the MPG. Although there was no difference in all-cause mortality between the two groups, the rapid progression group showed significantly higher rates of AVR. A high initial MPG (≥ 24 mmHg) was associated with progression that was eight times more rapid than that associated with a lower initial MPG (< 24 mmHg). When all analysis were performed with a subgroup of purely degenerative moderate AS, similar results were acquired with a cutoff initial MPG of 25 mmHg. Based on these findings, the initial MPG would be considered a predictor of rapid progression of MPG and future AVR in patients with moderate AS.

AS is considered a degenerative disease, with mechanical stress and valve thickening by lipid deposition and calcification contributing to its hemodynamic progression^18,19^. The natural history of AS varies widely among individuals, and little is known about the risk factors for its progression. Identification of these risk factors may be of great benefit for patients by allowing predictive insight in terms of intervention and disease management. Previous studies have identified several factors that are potentially associated with acceleration of AS. Demographic or clinical characteristics, including smoking, male sex, diabetes mellitus, hypertension, chronic renal dysfunction, and hyperlipidemia, show a positive correlation with progression of AS^20,21^. Ersboll et al. assessed hemodynamic progression in all stages of AS and found that an annual increase in the mean gradient of 7.1% in moderate AS, and age, sex, renal dysfunction, and hyperlipidemia were associated with MPG progression^22^. Yet, there were no studies evaluating the progression of moderate AS based on echocardiographic data-driven phenotyping.

To this date, there is a significant body of scientific literature that supports the idea that the severity of aortic stenosis at baseline can impact the progression rate. A review and meta-analysis suggested the relationship between baseline severity of the disease and AS progression, showing that baseline MPG was strongly associated with rapid progression^23^. Other initial parameters including peak velocity and AV calcification were also associated with progression rate. Nguyen et al. also revealed that AS tends to progress faster in individuals with a more severe initial disease state, regardless of other parameters^24^. The pathophysiology of AS initiation and progression include inflammatory response, calcification, and fibrotic changes of the valve. Mechanical stress damages the valve leaflets, while alteration in blood flow patterns and shear stress initiate valve stenosis^25^. Sun et al. showed that abnormal magnitude or frequency of shear stress altered the tissue expression of cytokines including BMP-4 and TGF-β1 and promote remodeling of extracellular matrix in porcine aortic valves^26^. After this inflammatory response, progression of the disease then is accelerated by fibrosis and calcification, which again leads to further changes in mechanical stress and blood flow, thus creating a vicious cycle. Taken together with our finding, the current evidence suggests that the baseline severity of stenosis may be a critical determinant of the progression rate.

In this study, we were able to perform data-driven phenotyping of moderate AS and classify it into two groups based on MPG progression with the use of longitudinal serial echocardiographic data. Interestingly, there was no significant difference in the clinical correlates among the 686 patients, except for atrial fibrillation and congestive heart failure, both of which were more prevalent in the slow progression group. However, there were significant between-group differences in all echocardiographic findings, with the main parameters used for AS evaluation (initial MPG, PPG, PV, and AV area) being more severe in the rapid progression group. As the grouping was based on MPG progression, we focused on the initial MPG at the time of diagnosis of moderate AS. Our findings suggest that a cut-off MPG value of 24 mmHg is a strong independent predictor of rapid progression of MPG. Due to the retrospective nature of this study, we cannot confirm if the two groups were totally distinct groups with different clinical characteristics or merely same group of patients captured in different time sequences during natural progression. Nonetheless, two discrete groups were identified based on initial MPG, implying the potential use for setting disease management and follow-up plans.

Recently, there have been attempts to identify trajectories in cardiovascular disease^27,28^, but no studies on the hemodynamic progression of moderate AS. Our results may assist in identifying the hemodynamic progression of moderate AS, providing the factors that may have an association with different progression patterns. For this purpose, we evaluated disease progression with AVR rate and also time to events, showing the significant difference between the trajectories. These findings may help us to prognosticate in patients with moderate AS and plan an appropriate follow-up. For example, patients with an initial MPG ≥ 24 mmHg would be more likely to be the rapid progression group and have a 51.6% risk of AVR within 5 years (Supplementary Table 4). Current guidelines recommend echocardiogram examination every 1–2 years for all patients with moderate AS^1^. Our finding suggests that there are two distinctive groups of hemodynamic progression, so the follow-up examination should be more personalized. The high initial MPG (≥ 24 mmHg) can be a good proxy for the subclassification of moderate AS; patients with initial MPG ≥ 24 mmHg may be informed of the chance of rapid progression and would benefit from at least 1-year follow-up regularly.

In this study, there was no difference in the mortality rate between the rapid progression group and slow progression group. A study by Strange et al. with a large cohort of AS and data of long-term survival showed that moderate AS was associated with high mortality rates similar to that of severe AS patients^6^. However, a more recent study by Coisne et al. demonstrated the survival outcomes in various grades of AS severity, showing a much lower mortality rate in patients with moderate AS than that of severe AS, while being only slightly higher than patients with mild AS^7^. The non-difference of mortality between the two groups in our study may be attributed to appropriate AVR in patients with progression to severe symptomatic AS. Therefore, the trajectory phenotyping would be mainly used to predict the timing of AVR; those with an MPG ≥ 24 mmHg would have a higher probability of requiring AVR in the future than those with a lower value.

Another point to discuss are the results from the subgroup of degenerative moderate AS patients. The main cause of AS in industrial countries is known to be degenerative calcification of the valve, while rheumatic valvular remains a common cause in developing countries^29,30^. With the overall development of countries worldwide and aging of the total population bringing more attention to degenerative AS, we analyzed all results in patients with a degenerative etiology only. The subgroup showed similar clinical trends and outcomes to those of the total group of moderate AS including all etiologies. There was a slight upward change in the cut-off initial MPG for group classification to 25 mmHg. However, almost all results remained unchanged, again demonstrating the difference in progression rate and AVR during follow-up with no significant difference in mortality between the rapid and slow progression groups.

### Limitations

This study has several limitations. First, it had a retrospective design and was performed at a single center; therefore, its findings might not be generalizable. Future prospective multicenter investigations may help to confirm our results. Second, some patients were lost to follow-up, which may have introduced a degree of bias. However, information on patient survival was complete because of linking of national mortality data, thereby providing reliable mortality data. The proportions of out-of-hospital deaths were similar in both groups, suggesting low possibility of bias (Supplementary Table 13). Despite that the cause of deaths was not provided, the use of all-cause mortality may have prevented the misclassification of causality^31^. Third, there may have been inter-observer variability in echocardiography performance and interpretation of the original TTE data. Finally, disease progression was based on MPG, with less focus on other echocardiographic parameters such as PPG, PV, and AV area. However, MPG represents hemodynamic severity of AS, also having independent prognostic values of disease progression^1,24^. Phenotyping of AS using additional measurements may help to identify more risk factors for progression of AS.

## Conclusion

By leveraging data-driven temporal phenotyping, we identified two distinct groups of patients with moderate AS: one characterized by slow progression and the other by rapid progression. A higher initial MPG (≥ 24 mmHg) was associated with rapid progression of AS and higher rates of AVR, indicating the predictive value of MPG in management of the disease.

## Supplementary Information

Supplementary Information.

## Data Availability

The datasets generated during and/or analyzed during the current study are available from the corresponding author on reasonable request.
