# Supplementary material for: Reclassification of moderate aortic stenosis based on data-driven phenotyping of hemodynamic progression
Source: Sci Rep. 2023 Apr 24;13:6694. doi: 10.1038/s41598-023-33683-1 (PMC10125992; doi:10.1038/s41598-023-33683-1)

**Supplemental Materials**

**Supplementary Table 1. Flow chart of model selection**

|  | **Description** | **Criteria for selection** |
| --- | --- | --- |
| **1** | Data preprocessing and sampling |  |
| **2** | Make several models assuming MPG progression trajectory | Univariate linear model  Univariate quadratic model  Multivariate linear model  Multivariate quadratic model |
| **3** | Assess the model adequacy and confirm the optimal number of classes in each model (K = 2~6). | BIC  Proportions per class  Posterior probability  Relative entropy  Clinical implementation |
| **4** | Confirm the clinical characterization and plausibility |  |

The Bayesian information criteria (BIC) is calculated as follows:

$$BIC= -2*LL+log\left( N \right)*k$$

where LL is the log-likelihood of the model, N is the number of examinations in the sample, and k is the number of parameters in the model. The relative entropy is a standardized version of entropy. The entropy is defined as follows:

$$E= -\sum_{i=1}^{N} \sum_{k=1}^{K} \hat{p}_{\mathrm{ik}}{\log\hat{p}}_{\mathrm{ik}}$$

where p is posterior probabilities. Relative entropy is defined as follows:

$$E_{k}=1- \frac{E}{N logK'}$$

where values close to 1 indicate lowest classification uncertainty.

**Supplementary Table 2. Model adequacy assessments of latent class trajectory models by K = 2~6**

| Model | K | BIC | Proportions per class % | Average posterior probability assignment | Relative entropy |
| --- | --- | --- | --- | --- | --- |
| Model 1 | 2 | 20716.42 | 44.6 : 55.4 | 0.76 : 0.91 | 0.51 |
| Model 2 | 3 | 20707.68 | 43.6 : 8.8: 47.7 | 0.79 : 0.80 : 0.79 | 0.54 |
| Model 3 | 4 | 20708.80 | 42.3 : 1.5 : 50.2 : 6.1 | 0.78 : 0.82 : 0.86 : 0.75 | 0.70 |
| Model 4 | 5 | 20725.05 | 1.5 : 50.9 : 19.5 : 20.4 : 7.7 | 0.79 : 0.85 : 0.69 : 0.70 : 0.79 | 0.66 |
| Model 5 | 6 | 20730.86 | 1.5 : 18.2 : 25.8 : 25.1 : 22.45 : 7.0 | 0.82 : 0.58 : 0.69 : 0.66 : 0.70 : 0.73 | 0.58 |

*BIC, Bayesian information criteria*

**Supplementary Table 3. Univariate Cox analysis for outcomes**

|  | **Death** | | **AVR** | |
| --- | --- | --- | --- | --- |
|  | **Hazard Ratio** (95% CI) | ***P* value** | **Hazard Ratio** (95% CI) | ***P* value** |
| **Group**  (Ref = Group 1) | 0.80 (0.59-1.1) | 0.169 | 3.50 (2.49-4.93) | <.001 |
| **Sex**  (Ref = Men) | 0.67 (0.49-0.92) | 0.012 | 0.86 (0.67-1.11) | 0.242 |
| **Age** (10 y) | 2.20 (1.83-2.65) | <.001 | 1.12 (1.00-1.25) | 0.044 |
| **Hypertension** | 2.20 (1.55-3.11) | <.001 | 1.20 (0.93-1.55) | 0.168 |
| **Dyslipidemia** | 1.12 (0.95-1.32) | 0.181 | 0.94 (0.77-1.15) | 0.566 |
| **Diabetes** | 2.48 (1.82-3.38) | <.001 | 1.19 (0.90-1.56) | 0.218 |
| **PAOD** | 2.46 (1.46-4.14) | <.001 | 2.07 (1.06-4.02) | 0.032 |
| **Stroke** | 1.76 (1.15-2.69) | 0.009 | 0.65 (0.40-1.06) | 0.086 |
| **Chronic kidney disease** | 5.29 (3.79-7.39) | <.001 | 1.56 (1.08-2.25) | 0.018 |
| **COPD** | 2.04 (1.13-3.68) | 0.018 | 1.38 (0.60-3.19) | 0.454 |
| **Coronary artery disease** | 1.49 (1.07-2.06) | 0.017 | 1.11 (0.83-1.49) | 0.467 |
| **Coronary artery bypass graft** | 2.10 (1.30-3.38) | 0.002 | 1.00 (0.55-1.83) | 0.996 |
| **Congestive heart failure** | 1.59 (1.00-2.52) | 0.049 | 1.08 (0.68-1.73) | 0.735 |
| **Atrial fibrillation** | 1.15 (0.82-1.6) | 0.422 | 0.71 (0.52-0.97) | 0.032 |
| **Rheumatic heart disease** | 0.37 (0.23-0.58) | <.001 | 0.72 (0.54-0.98) | 0.035 |
| **Bicuspid aortic valve** | 0.36 (0.19-0.68) | 0.002 | 0.89 (0.61-1.28) | 0.521 |
| **Aortic regurgitation** | 0.40 (0.25-0.64) | <.001 | 0.83 (0.63-1.10) | 0.203 |
| **Initial MPG** | 0.98 (0.96-1.01) | 0.260 | 1.07 (1.05-1.09) | <.001 |

**Supplementary Table 4. Cumulative outcome frequency**

|  | **1 year** | | **2 years** | | **5 years** | | **Total** | |
| --- | --- | --- | --- | --- | --- | --- | --- | --- |
|  | **Slow** | **Rapid** | **Slow** | **Rapid** | **Slow** | **Rapid** | **Slow** | **Rapid** |
| **AVR** | 1 (0.3) | 6 (1.6) | 9 (2.9) | 29 (7.6) | 34 (11.1) | 142 (37.4) | 41 (13.4) | 196 (51.6) |
| **Surgical** | 0 (0.0) | 5 (1.3) | 7 (2.3) | 20 (5.3) | 25 (8.2) | 99 (26.1) | 31 (10.1) | 137 (36.1) |
| **TAVR** | 1 (0.3) | 1 (0.3) | 2 (0.7) | 9 (2.4) | 9 (2.9) | 43 (11.3) | 10 (3.3) | 59 (15.5) |
| **Mortality** | 7 (2.3) | 3 (0.8) | 20 (6.5) | 20 (5.3) | 54 (17.6) | 69 (18.2) | 69 (22.5) | 95 (25.0) |

* Freq. (percentage) at respective year

AVR, aortic valve replacement; TAVR, transcatheter aortic valve replacement

**Supplementary Table 5. Probability of being allocated to the rapid progression group in univariate logistic regression**

|  | **Odd Ratio** (95% CI) | ***P* value** |
| --- | --- | --- |
| Initial MPG  (Ref: Initial MPG <24 mmHg) | 6.36 (3.97-10.30) | <.001 |
| Sex  (Ref: Men) | 0.93 (0.61-1.43) | 0.749 |
| Age (per 10 years) | 1.10 (0.92-1.31) | 0.294 |
| Hypertension | 0.82 (0.52-1.28) | 0.390 |
| Dyslipidemia | 0.78 (0.51-1.13) | 0.235 |
| Diabetes | 0.78 (0.50-1.23) | 0.284 |
| PAOD | 1.57 (0.56-5.09) | 0.413 |
| Stroke | 0.67 (0.32-1.38) | 0.267 |
| Chronic kidney disease | 0.78 (0.45-1.37) | 0.389 |
| COPD | 0.94 (0.31-3.07) | 0.910 |
| Coronary artery disease | 0.88 (0.50-1.34) | 0.603 |
| Coronary artery bypass graft | 0.92 (0.32-2.75) | 0.871 |
| Congestive heart failure | 0.52 (0.26-1.02) | 0.058 |
| Atrial fibrillation | 0.60 (0.37-0.99) | 0.045 |
| Rheumatic heart disease | 0.73 (0.43-1.24) | 0.247 |
| Bicuspid aortic valve | 1.56 (0.77-3.33) | 0.232 |
| Aortic regurgitation | 1.04 (0.62-1.75) | 0.884 |

**Supplementary Table 6. Risk of each outcome in the two trajectory groups and age interaction.**

| **Parameters** | **Hazard ratio (95% CI)^*^** | ***P for interaction*** |
| --- | --- | --- |
|  |  |  |
| **AVR** | 0.73 (0.56-0.94) | 0.015 |
| **Surgical** | 0.64 (0.51-0.80) | <0.001 |
| **Transcatheter** | 1.27 (0.48-3.36) | 0.635 |
| **Mortality** | 1.18 (0.81-1.70) | 0.393 |

*AVR, aortic valve replacement; CI, confidence interval***Supplementary Table 7. Risk of each outcome in the two trajectory groups and sex interaction.**

| **Parameters** | **Hazard ratio (95% CI)^*^** | ***P for interaction*** |
| --- | --- | --- |
|  |  |  |
| **AVR** | 1.93 (0.97-3.84) | 0.062 |
| **Surgical** | 1.46 (0.64-3.33) | 0.369 |
| **Transcatheter** | 1.89 (0.44-8.19) | 0.395 |
| **Mortality** | 0.74 (0.38-1.44) | 0.374 |

*AVR, aortic valve replacement; CI, confidence interval*

**Supplementary Table 8. Probability of being allocated to the rapid progression group in multivariate logistic regression**

|  | **Odd Ratio** (95% CI) | ***P* value** |
| --- | --- | --- |
| Initial MPG  (Ref: Initial MPG <24 mmHg) | 6.29 (3.92 – 10.24) | <.001 |
| Atrial fibrillation | 0.64 (0.37 – 1.10) | 0.106 |

*MPG, mean pressure gradient; CI, confidence interval*

*Covariates showing significant *P* values within 90% CI in univariate analysis were included for multivariate analysis.

**Supplementary Table 9. Baseline demographic and clinical characteristics in degenerative moderate aortic stenosis patients without other valve diseases**

| **Characteristic** | **Overall**  **(n=311)** | **Slow progression group**  **(n=231)** | **Rapid progression group**  **(n=80)** | ***P-*value** |
| --- | --- | --- | --- | --- |
| Age, years | 73.2 (9.8) | 73.3 (9.9) | 72.7 (9.5) | 0.616 |
| Female (%) | 143 (46.0) | 107 (46.3) | 36 (45.0) | 0.838 |
| BMI >25 kg/m^2^ (%) | 134 (43.1) | 96 (41.6) | 38 (47.5) | 0.355 |
| Comorbid conditions (%) |  |  |  |  |
| Hypertension | 243 (78.1) | 183 (79.2) | 60 (75.0) | 0.431 |
| Dyslipidemia | 147 (47.3) | 119 (51.5) | 28 (35.0) | 0.011 |
| Diabetes | 139 (44.7) | 115 (49.8) | 24 (30.0) | 0.002 |
| PAOD | 22 (7.1) | 16 (6.9) | 6 (7.5) | 0.863 |
| Stroke | 30 (9.6) | 23 (10.0) | 7 (8.8) | 0.753 |
| Chronic kidney disease | 75 (24.1) | 59 (25.5) | 16 (20.0) | 0.318 |
| COPD | 17 (5.5) | 14 (6.1) | 3 (3.8) | 0.433 |
| Coronary artery disease | 113 (36.3) | 90 (39.0) | 23 (28.8) | 0.102 |
| Coronary artery bypass graft | 20 (6.4) | 18 (7.8) | 2 (2.5) | 0.096 |
| Congestive heart failure | 34 (10.9) | 31 (13.4) | 3 (3.8) | 0.017 |
| Atrial fibrillation | 50 (16.1) | 37 (16.0) | 13 (16.3) | 0.961 |
| Aortic regurgitation | 28 (9.0) | 23 (10.0) | 5 (6.3) | 0.318 |
| Reason for echocardiography |  |  |  |  |
| Symptomatic* | 105 (33.8) | 85 (36.8) | 20 (25.0) | 0.054 |
| Routine follow-up for known mild AS | 53 (17.0) | 30 (13.0) | 23 (28.8) | 0.001 |
| Systematic disease or other cardiac disease^†^ | 148 (47.6) | 113 (48.9) | 35 (43.8) | 0.425 |
| Evaluation for non-cardiac procedures | 5 (1.6) | 3 (1.3) | 2 (2.5) | 0.462 |
| Echocardiographic findings |  |  |  |  |
| Mean interscan interval, years | 1.0 ± 0.6 | 1.0 ± 0.6 | 1.0 ± 0.6 | 0.495 |
| Initial MPG, mmHg | 25.1 ± 5.0 | 23.2 ± 3.0 | 30.6 ± 5.3 | <.001 |
| MPG mean progression rate, mmHg/year | 2.3 ± 5.8 | 1.5 ± 5.6 | 4.6 ± 5.8 | <.001 |
| Initial PPG, mmHg | 44.4 ± 9.2 | 41.1 ± 6 | 53.9 ± 10.0 | <.001 |
| Initial peak velocity, m/s | 3.3 ± 0.3 | 3.2 ± 0.2 | 3.7 ± 0.2 | <.001 |
| Initial AVA by CE, cm^2^ | 1.2 ± 0.2 | 1.2 ± 0.2 | 1.1 ± 0.2 | <.001 |
| LVOT VTI, cm | 23.5 ± 5.5 | 23.5 ± 5.6 | 23.6 ± 5.2 | 0.846 |
| LVEF (%) | 46.1 (11.4) | 44.9 (11.5) | 53.8 (7.9) | <.001 |
| LV end-diastolic dimension, mm | 49.4 ± 6.5 | 49.4 ± 6.8 | 49.3 ± 5.5 | 0.814 |
| LV end-systolic dimension, mm | 32.3 ± 7.0 | 32.6 ± 7.6 | 31.6 ± 4.7 | 0.192 |

AVA, aortic valve area; BMI, body mass index; CE, continuity equation; COPD, chronic obstructive pulmonary disease; LV, left ventricular; LVEF, left ventricular ejection fraction; LVOT, left ventricular outflow tract; MPG, mean pressure gradient; PAOD, peripheral arterial occlusive disease; PPG, peak pressure gradient; VTI, time–velocity integral

*Symptomatic: Chest pain, dyspnea, syncope, dizziness, edema, general weakness

^†^Systematic disease or other cardiac disease: Other cardiac disease (arrythmia, coronary artery disease, hypertrophic cardiomyopathy, infective endocarditis), systematic disease (chronic kidney disease, intracranial hemorrhage, sepsis)

**Supplementary Table 10. Incidence rates and risk of each outcome in the two trajectory groups of degenerative moderate aortic stenosis patients without other valve diseases**

| **Parameters** | **Incidence rate (CI)** | | **Hazard ratio (95% CI)**^*^ |  |
| --- | --- | --- | --- | --- |
|  | **Slow progression group** | **Rapid progression group** |  | ***P*-value** |
| **AVR** | 6.1 (4.5-8.1) | 16.7 (12.1-22.5) | 3.0 (1.9-4.6)^†^ | <.001 |
| **Surgical** | 2.3 (1.4-3.7) | 9.2 (6.0-13.4) | 4.7 (2.4-9.0)^‡^ | <.001 |
| **Transcatheter** | 3.5 (2.3-5.1) | 5.3 (3.1-8.5) | 2.0 (1.1-3.7)^§^ | 0.026 |
| **Mortality** | 6.8 (5.1-8.8) | 6.4 (4.0-9.6) | 1.4 (0.9-2.3)^∥^ | 0.184 |

AVR, aortic valve replacement; CI, confidence interval

^*^ Hazard ratios for rapid progression group vs slow progression group

^†^ Adjusted with group type, aortic regurgitation

^‡^ Adjusted with group type, age (10 years), dyslipidemia, chronic obstruction pulmonary disease, coronary artery bypass graft

^§^ Adjusted with group type, age (10 years), hypertension, dyslipidemia, diabetes, chronic kidney disease, aortic regurgitation

^∥^ Adjusted with group type, age (10 years), diabetes, chronic kidney disease, coronary artery bypass graft

**Supplementary Table 11. Univariate Cox analysis for outcomes in degenerative moderate aortic stenosis patients without other valve diseases**

|  | **Death** | | **AVR** | |
| --- | --- | --- | --- | --- |
|  | **Hazard Ratio** (95% CI) | ***P* value** | **Hazard Ratio** (95% CI) | ***P* value** |
| **Group**  (Ref = Group 1) | 0.95 (0.58-1.56) | 0.853 | 2.73 (1.77-4.21) | <.001 |
| **Sex**  (Ref = Men) | 0.65 (0.40-1.06) | 0.085 | 0.79 (0.50-1.26) | 0.320 |
| **Age** (10 y) | 2.28 (1.56-3.33) | <.001 | 1.11 (0.871-1.42) | 0.392 |
| **Hypertension** | 1.83 (0.95-3.51) | 0.071 | 1.26 (0.71-2.22) | 0.432 |
| **Dyslipidemia** | 1.35 (0.84-2.15) | 0.213 | 0.99 (0.62-1.59) | 0.972 |
| **Diabetes** | 2.92 (1.77-4.82) | <.001 | 1.34 (0.83-2.17) | 0.234 |
| **PAOD** | 1.43 (0.73-2.79) | 0.294 | 1.75 (0.62-4.96) | 0.289 |
| **Stroke** | 1.07 (0.48-2.42) | 0.862 | 0.73 (0.32-1.66) | 0.453 |
| **Chronic kidney disease** | 4.06 (2.52-6.55) | <.001 | 2.35 (1.35-4.06) | 0.002 |
| **COPD** | 1.66 (0.68-4.05) | 0.269 | 1.35 (0.45-4.03) | 0.588 |
| **Coronary artery disease** | 1.14 (0.71-1.84) | 0.584 | 1.02 (0.63-1.67) | 0.923 |
| **Coronary artery bypass graft** | 2.01 (1.14-3.54) | 0.016 | 1.1 (0.37-3.32) | 0.866 |
| **Congestive heart failure** | 1.18 (0.49-2.85) | 0.706 | 1.77 (0.90-3.48) | 0.100 |
| **Atrial fibrillation** | 1.73 (0.96-3.09) | 0.067 | 0.94 (0.43-2.03) | 0.867 |
| **Aortic regurgitation** | 0.37 (0.12-1.29) | 0.118 | 1.27 (0.70-2.33) | 0.433 |

**Supplementary Table 12. Probability of being allocated to the rapid progression group in univariate logistic regression of degenerative moderate aortic stenosis patients without other valve diseases**

|  | **Odd Ratio** (95% CI) | ***P* value** |
| --- | --- | --- |
| Initial MPG  (Ref: Initial MPG <25 mmHg) | 16.70 (5.24-77.40) | <.001 |
| Sex  (Ref: Men) | 0.95 (0.37-2.38) | 0.910 |
| Age (per 10 years) | 0.94 (0.61-1.52) | 0.786 |
| Hypertension | 0.79 (0.29-2.51) | 0.659 |
| Dyslipidemia | 0.51 (0.19-1.28) | 0.163 |
| Diabetes | 0.43 (0.15-1.12) | 0.097 |
| PAOD | 1.09 (0.12-4.91) | 0.923 |
| Stroke | 0.87 (0.12-3.50) | 0.862 |
| Chronic kidney disease | 0.73 (0.21-2.09) | 0.584 |
| COPD | 0.60 (0.02-3.89) | 0.675 |
| Coronary artery disease | 0.63 (0.21-1.65) | 0.297 |
| Coronary artery bypass graft | 0.30 (0.00-2.41) | 0.408 |
| Congestive heart failure | 0.25 (0.00-1.5) | 0.243 |
| Atrial fibrillation | 1.02 (0.25-3.17) | 0.978 |
| Aortic regurgitation | 0.60 (0.05-2.82) | 0.593 |

**Supplementary Table 13. In-hospital mortality and mortality data from the Korean Ministry of the Interior and Safety**

|  | Slow progression group  (n=306) | Rapid progression group  (n=380) | Total  (n=686) |
| --- | --- | --- | --- |
| In-hospital deaths | 32 | 47 | 79 |
| KMIS deaths | 69 | 95 | 164 |
| Information loss * (%) | 37 (53.6) | 48 (50.5) | 85 (51.8) |

*KMIS, Korean Ministry of the Interior and Safety*

* Information loss = KMIS deaths − In-hospital deaths

**Supplementary Figure 1. Identification of the study population**

**
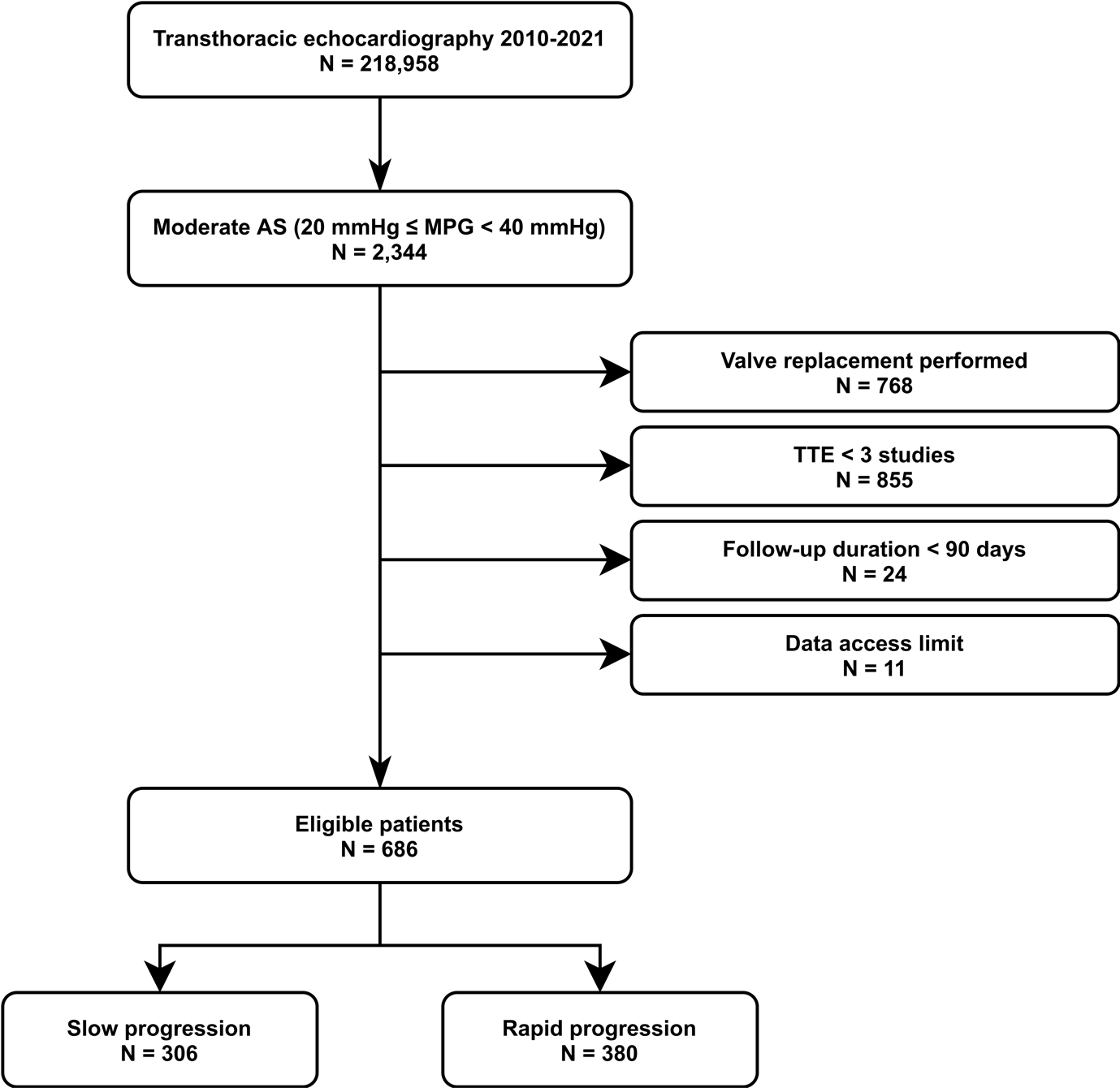
**

*AS, aortic stenosis; MPG, mean pressure gradient; TTE, transthoracic echocardiography*

**Supplementary Figure 2. Classification of patients with moderate aortic stenosis using LTCM based on progression of MPG in the model verify validation group. (A) Raw LCTM results. (B) Smoothed LCTM results identify two distinct trajectories.**


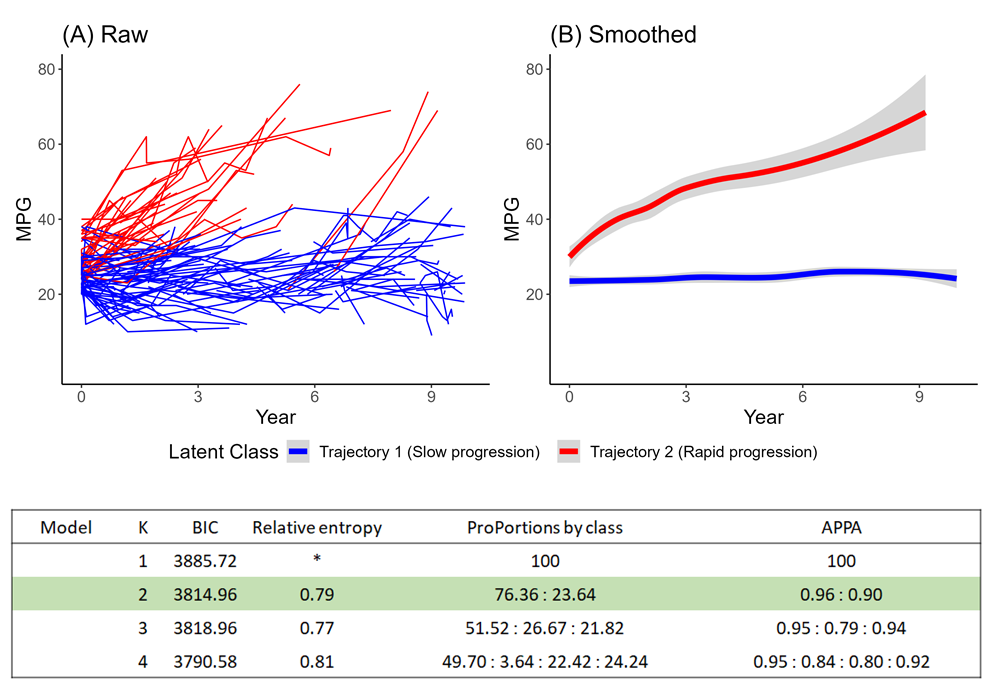


**Supplementary Figure 3. Kaplan–Meier curves of AVR (surgical and TAVR) according to groups**


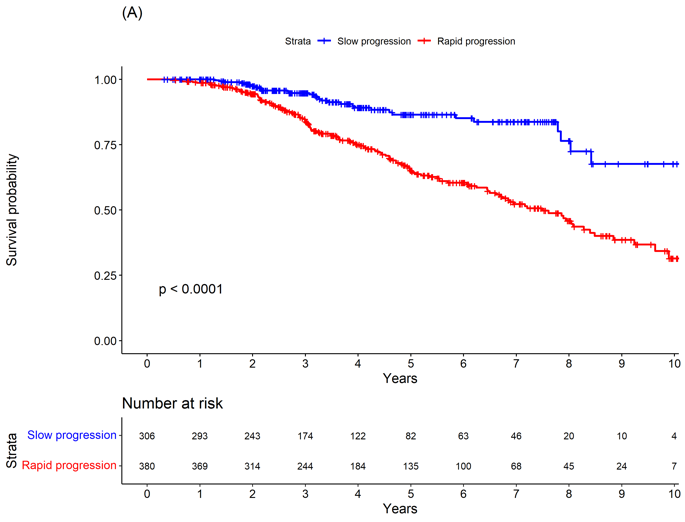

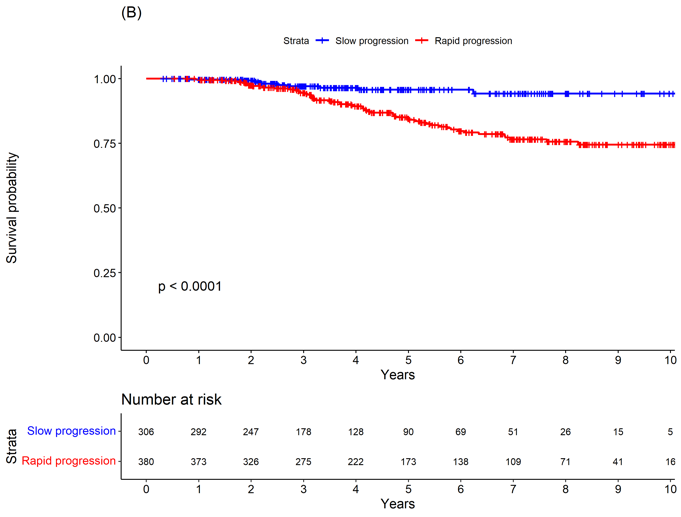


*AVR, aortic valve replacement; TAVR, transcatheter aortic valve replacement*

**Supplementary Figure 4. (A) Receiver operating characteristic for mean pressure gradient (MPG) as a predictor for rapid progression group allocation and (B) density plot of baseline MPG in the two groups**

*
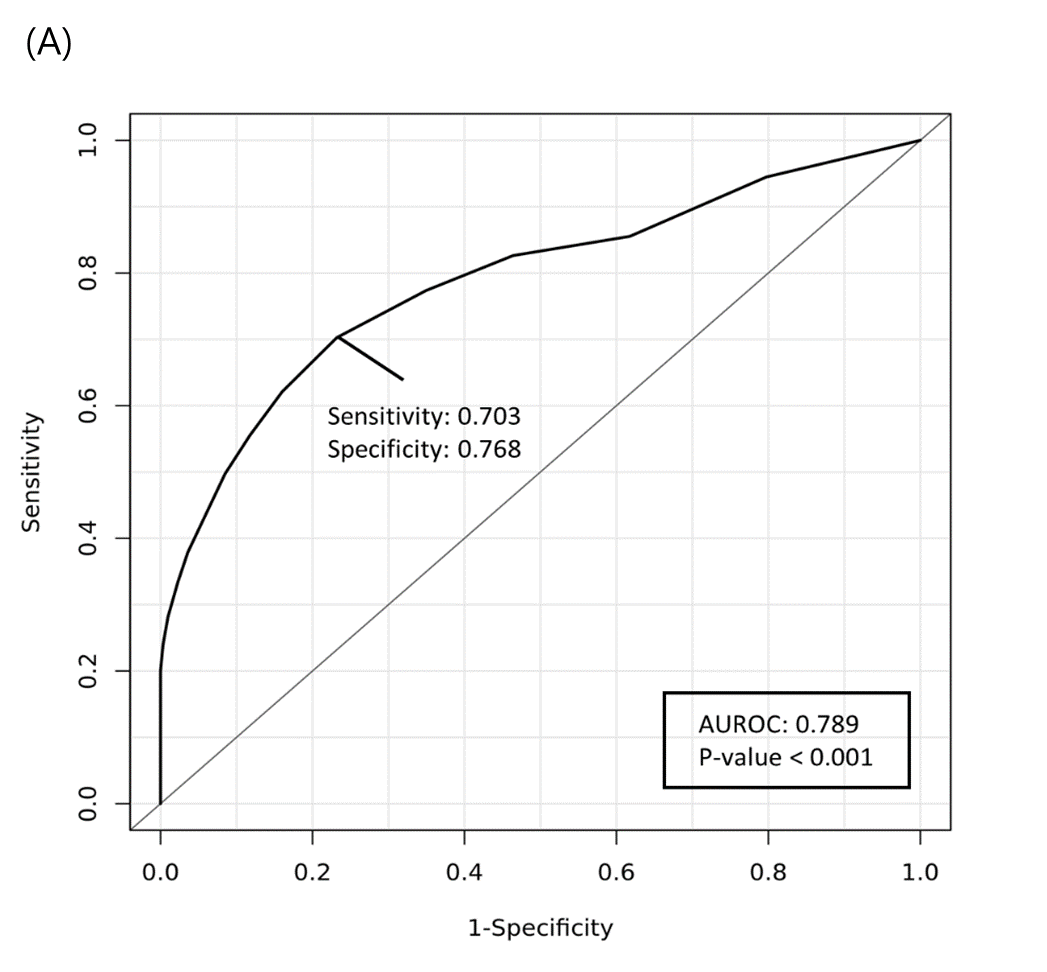
*
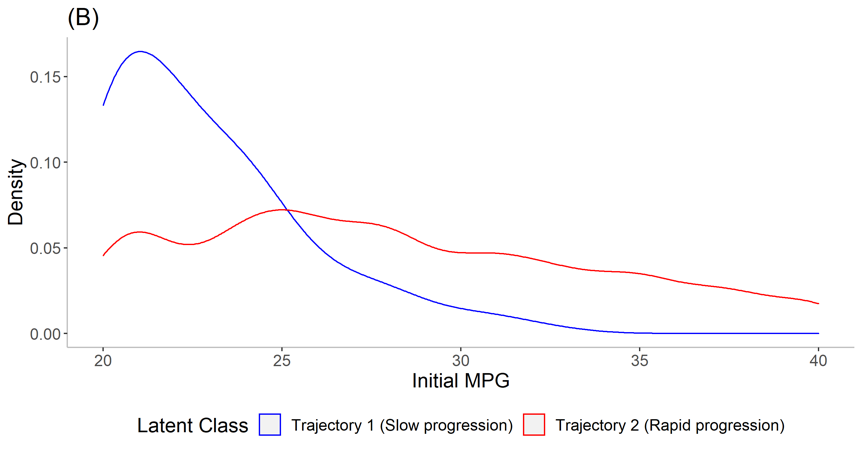


**Supplementary Figure 5. Receiver operating characteristic for mean pressure gradient (MPG) as a predictor for rapid progression group allocation in the** **model validation group**

**
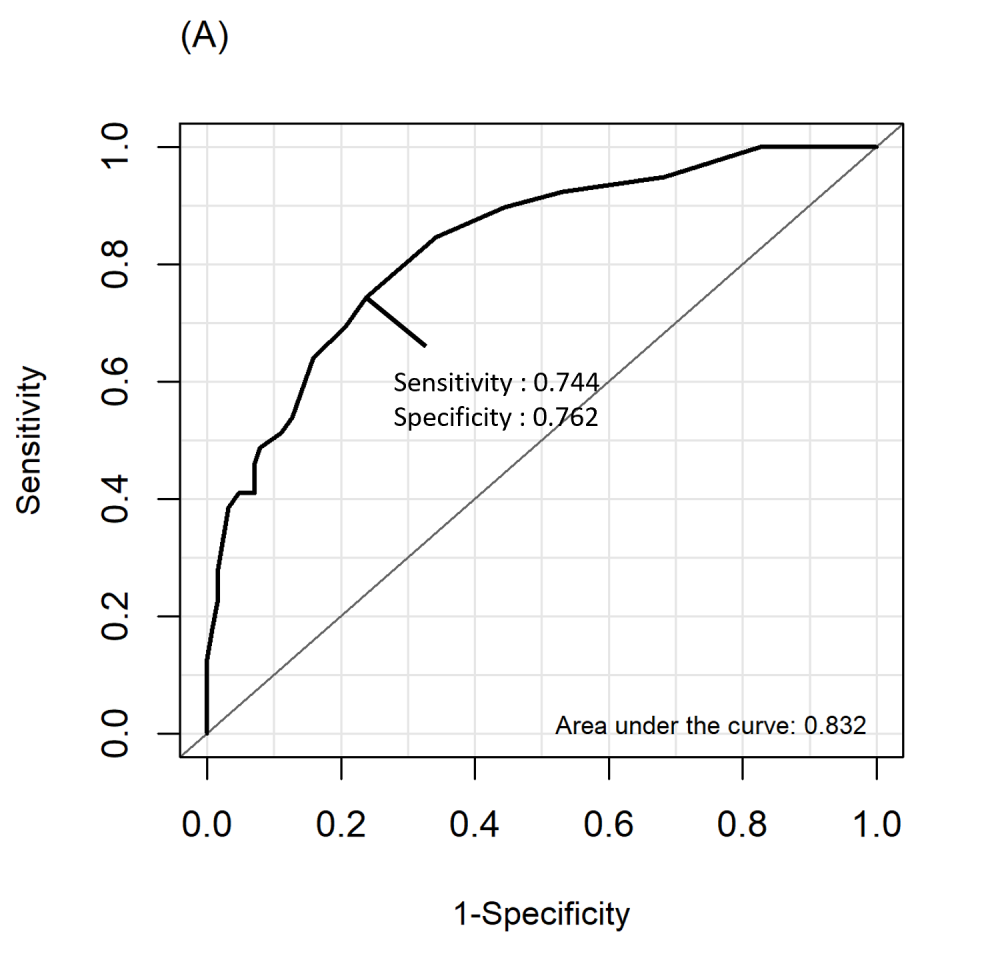
**

**Supplementary Figure 6. Cubic spline curve showing association of MPG with risks for (A) AVR and (B) death**

**
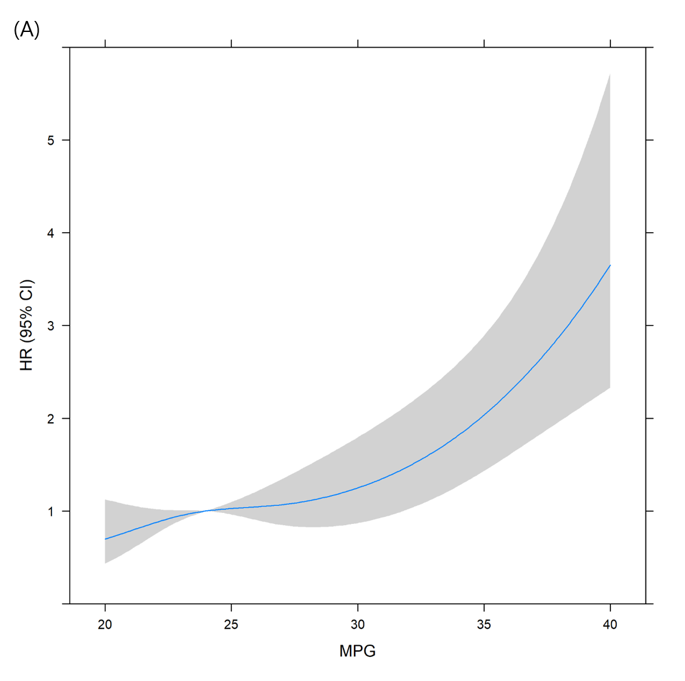

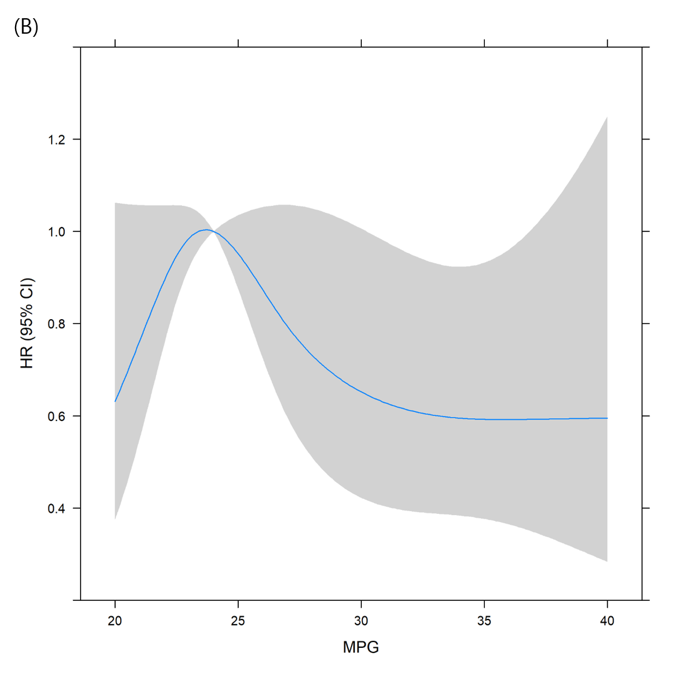
**

*AVR, aortic valve replacement; CI, confidence interval; HR, hazard ratio; MPG, mean pressure gradient

**Supplementary Figure 7.** **Classification of patients with degenerative moderate aortic stenosis without other valve diseases using LTCM based on progression of MPG. (A) Raw LCTM results. (B) Smoothed LCTM results identify two distinct trajectories. (C) Receiver operating characteristic for MPG as a predictor for rapid progression group allocation and (D) density plot of baseline MPG in the two groups.**

LCTM, latent class trajectory modeling; MPG, mean pressure gradient


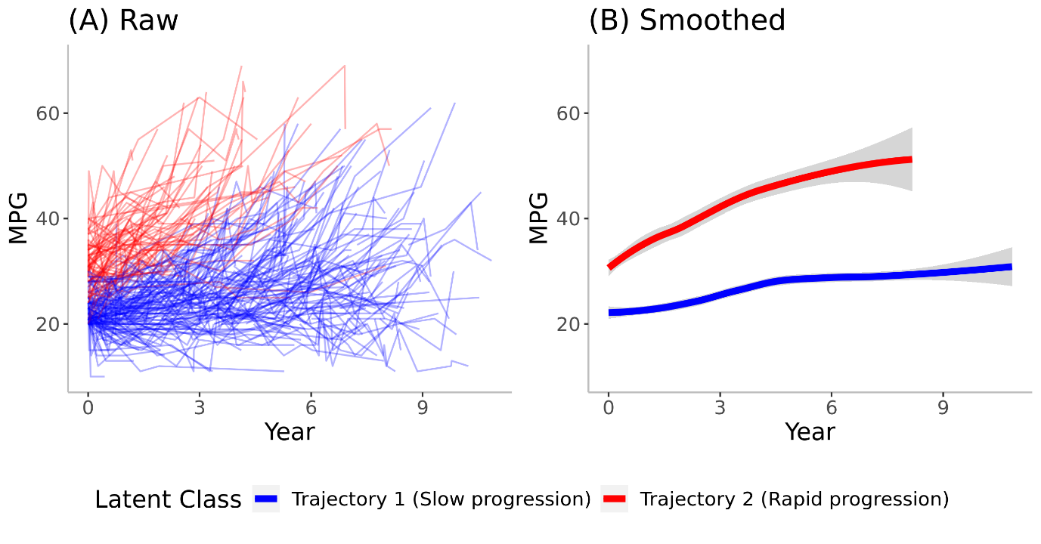
*
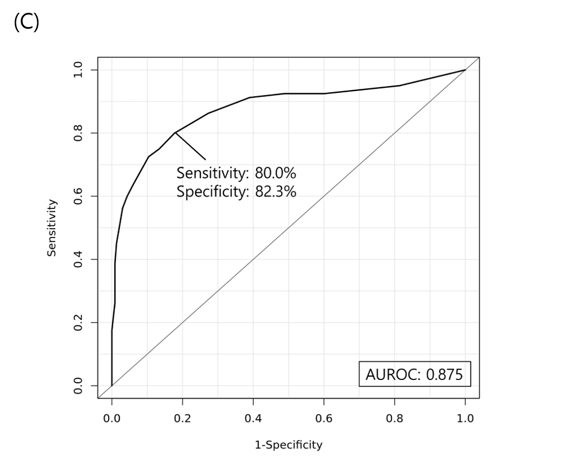

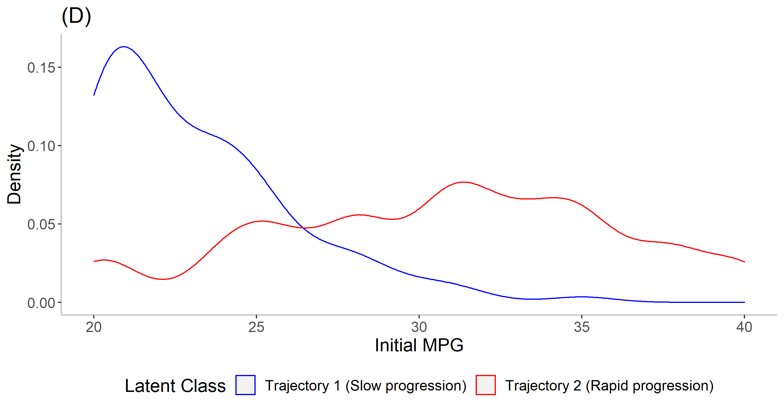
*

**Supplementary Figure 8. Kaplan–Meier curves for (A) aortic valve replacement and (B) death eFigure 5. Kaplan–Meier curves for (A) aortic valve replacement and (B) death according to progression group in degenerative moderate aortic stenosis patients without other valve diseases.**


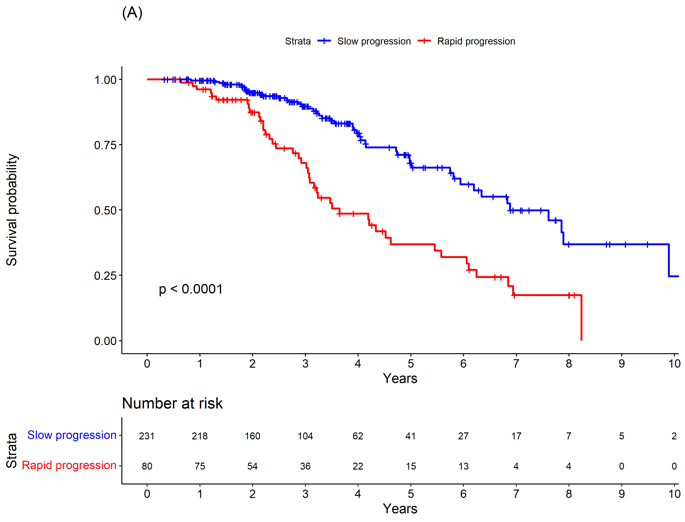

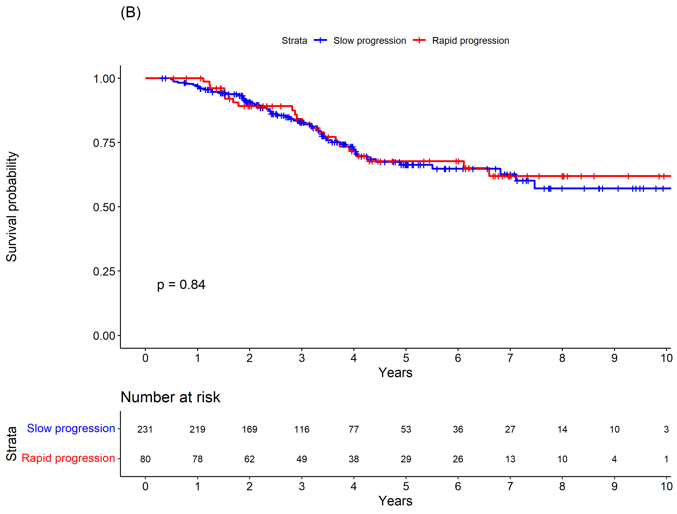

Supplement: Supplementary file 1 — Supplementary Information. [file 41598_2023_33683_MOESM1_ESM.docx]
